# Comprehensive characterization of extracellular matrix-related genes in PAAD identified a novel prognostic panel related to clinical outcomes and immune microenvironment: A silico analysis with *in vivo* and vitro validation

**DOI:** 10.3389/fimmu.2022.985911

**Published:** 2022-10-13

**Authors:** Xu Chen, Qihang Yuan, Jifeng Liu, Shilin Xia, Xueying Shi, Yuxin Su, Zhizhou Wang, Shuang Li, Dong Shang

**Affiliations:** ^1^ Department of General Surgery, First Affiliated Hospital of Dalian Medical University, Dalian, China; ^2^ Laboratory of Integrative Medicine, First Affiliated Hospital of Dalian Medical University, Dalian, China; ^3^ Institute (College) of Integrative Medicine, Dalian Medical University, Dalian, China; ^4^ Department of Medical Imaging, Cardiovascular Research Institute, Northern Theater Command General Hospital, Shenyang, China

**Keywords:** pancreatic adenocarcinoma, ECM receptor interaction, molecular classification, prognosis signature, chemotherapy sensitivity

## Abstract

The extracellular matrix (ECM) is a vital component of the tumor microenvironment, which interplays with stromal and tumor cells to stimulate the capacity of cancer cells to proliferate, migrate, invade, and undergo angiogenesis. Nevertheless, the crucial functions of ECM-related genes (ECMGs) in pancreatic adenocarcinoma (PAAD) have not been systematically evaluated. Hence, a comprehensive evaluation of the ECMGs is required in pan-cancer, especially in PAAD. First, a pan-cancer overview of ECMGs was explored through the integration of expression profiles, prognostic values, mutation information, methylation levels, and pathway-regulation relationships. Seven ECMGs (i.e. LAMB3, LAMA3, ITGB6, ITGB4, ITGA2, LAMC2, and COL11A1) were identified to be hub genes of PAAD, which were obviously up-regulated in PAAD and considerably linked to tumor stage as well as prognosis. Subsequently, patients with PAAD were divided into 3 clusters premised on ECMG expression and ECM scores. Cluster 2 was the subtype with the best prognosis accompanied by the lowest ECM scores, further verifying ECM’s significant contribution to the pathophysiological processes of PAAD. Significant differences were observed for oncogene and tumor suppressor gene expression, immune microenvironment, and chemotherapy sensitivity across three ECM subtypes. After applying a variety of bioinformatics methods, a novel and robust ECM-associated mRNA-lncRNA-based prognostic panel (ECM-APP) was developed and validated for accurately predicting clinical outcomes of patients with PAAD. Patients with PAAD were randomly categorized into the train, internal validation, and external validation cohorts; meanwhile, each patient was allocated into high-risk (unfavorable prognosis) and low-risk (favorable prognosis) populations premised on the expression traits of ECM-related mRNAs and lncRNAs. The discrepancy in the tumor mutation burden and immune microenvironment might be responsible for the difference in prognoses across the high-risk and low-risk populations. Overall, our findings identified and validated seven ECMGs remarkably linked to the onset and progression of PAAD. ECM-based molecular classification and prognostic panel aid in the prognostic assessment and personalized intervention of patients with PAAD.

## Introduction

Pancreatic adenocarcinoma (PAAD) is among the most prevalent digestive tract malignancy, which is recognized as the “king of cancers” with increasing morbidity in the whole world (1). PAAD is a deadly neoplasm and a defiant public health problem globally. As a result of the absence of obvious clinical manifestations in the preliminary phase of PAAD, 90% of patients have already advanced to metastasis during diagnosis, and as a consequence, they miss the chance for surgical therapy. At the same time, PAAD has a certain degree of resistance to radiotherapy, molecular targeted therapy, and chemotherapy which leads to PAAD being a tumor with a very high mortality rate. Despite the ongoing advancement in the early diagnosis and treatment of PAAD, the five-year survival is only around 9% for patients with this cancer (2). Therefore, developing and validating new prognostic signatures to improve the prediction of the clinical outcomes and chemotherapy sensitivity of PAAD patients is still urgently required.

The tumor microenvironment (TME) performs a critical function in the process of carcinogenesis and cancer progression. In addition, the extracellular matrix (ECM) is a vital component of the TME. The ECM not only helps cells by providing them with mechanical and physical support but also helps cells communicate with one another *via* the exchange of biochemical signals (3). Through interactions with stromal and tumor cells, the ECM helps cancer cells to proliferate, migrate, invade, underwent angiogenesis, and evade the immune system (4, 5). Accordingly, the ECM performs an integral function in the regulation of carcinogenesis. Excessive ECM deposition is recognized as one of the markers of cancer interaction that is associated with an unfavorable prognosis for the patient (6–9). Furthermore, the EMC supplies cancer cells with persistent proliferative signals, including chemokines and growth factors, which may protect cancer cells from growth inhibitors while also acting as a diffusion barrier for traditional anti-cancer medications (10, 11). Moreover, to the best of our knowledge, the ECM-related genes (ECMGs) have not been discovered to predicate clinical outcomes and chemotherapeutic strategies in patients with PAAD. Thus, the development of the PAAD risk stratification tool using ECMs is promising.

In this research, we comprehensively evaluated the expression levels and genomic variations of the ECMGs across different types of cancer. Through combining in silico analysis with *in vitro* validation, seven ECMGs (i.e. LAMA3, ITGB6, ITGB4, ITGA2, LAMC2, and COL11A1) were confirmed to be considerably up-modulated in pancreatic cancer cell lines and clinical samples and were strongly associated with the prognosis and tumor stage of the patients. Based on ECM scores and ECMG expression, we categorized PAAD patients into three distinct types and evaluated their relationships with prognosis, immune microenvironments, and drug sensitivities. We further established and verified a novel and independent prognostic panel premised on ECM-related mRNAs and lncRNAs. Overall, our new findings might lay the foundation for a deeper comprehension of the pathophysiologic mechanisms of PAAD, decision-making in the clinical setting, and individualized treatment.

## Materials and methods

### Pan-cancer landscape of ECM-related genes

First, 84 ECMGs were obtained based on the following dataset from the Molecular Signatures Database (MSigDB): KEGG_ECM_RECEPTOR_INTERACTION. Although many ECMGs have been explored in tumors, pan-cancer characterization of ECMGs is not well summarized. Thus, intensive exploration of the contributions of these genes in a wide range of human malignancies from the perspective of expression traits, prognostic values, methylation, mutation, and pathways would therefore be highly warranted. The CNV and SNV data that were generated from the TCGA database were processed and presented utilizing heatmaps to provide a pan-cancer overview of variations of ECMGs (12). Furthermore, a pan-cancer assessment of methylation levels and differential mRNA expression were performed. In addition to this, a univariate Cox regression analysis between mRNA expression and overall survival (OS) was carried out to determine the value of ECMGs as a prognostic marker in diverse malignancies. R and TBtools were used to perform all of these analyses similarly with our previous published articles (13).

To unveil the differential role of pathways influenced by ECMGs in human multiple cancers, single sample gene set enrichment analysis (ssGSEA) was utilized to compute pathway scores in each sample of each tumor. Gene set enrichment analysis (GSEA) was employed to examine the differences in pathway activity using the transcriptomes of two tumor groups with the bottom and top 30% of pathway scores (14).

### Identification and validation of hub ECMGs related to the onset and advancement of PAAD

In this section, we devoted our attention to PAAD to conduct an in-depth and comprehensive investigation. The transcriptome data and clinical data of PAAD and normal pancreas tissues were acquired from Gene Expression Omnibus (GEO), Genotype-Tissue Expression (GTEx), International Cancer Genome Consortium (ICGC), and the Cancer Genome Atlas (TCGA) databases. After log2 transformation and batch correction, 178 PAAD and 171 normal pancreas samples in the GTEx and TCGA cohorts, 45 PAAD and 45 normal pancreas samples in the GSE28735 cohort, 69 PAAD and 61 normal pancreas samples in the GSE62452 cohort, and 82 PAAD samples in the ICGC database were obtained for further analysis.

To determine ECMGs that are expressed differentially between PAAD samples and normal pancreatic tissues, the ‘limma’ package in R was employed based on the cohorts of GSE28735, GSE62452, TCGA, and GTEx. The prognostic significance of differentially expressed ECMGs was examined utilizing univariate Cox regression analysis as well as Kaplan-Meier survival analysis. Gene Expression Profiling Interactive Analysis (GEPIA) platform was applied to evaluate the significant association of the above genes with tumor stage (15, 16). Generally speaking, the differentially expressed genes considerably linked to tumor prognosis and stage were often regarded as the hub genes of the onset and progression of tumors. Thus, we further intended to verify the results of differential expression of the above hub genes across PAAD and normal pancreas samples through immunohistochemistry (IHC) and quantitative real-time PCR (qRT-PCR).

#### Cell culture

The American Type Culture Collection (ATCC, Manassas, VA, USA) supplied the HPDE6-C7 normal human pancreatic ductal cell line. Our research laboratory provided the following human pancreatic cancer cell lines: PANC-1, CFPAC-1, and Bxpc-3. Cells of the HPDE6-C7, BxPC-3, and Panc-1 lines were incubated in Dulbecco’s modified Eagle’s medium (DMEM) (Gibco, USA) supplemented with 10% fetal bovine serum (FBS) (Gibco, USA) in line with the guidance provided by the ATCC. CF-PAC1 cells were incubated in Iscove’s Modified Dulbecco medium (IMDM) mixed with 10% FBS (Procell, China). All of the cell lines were grown at 37 °C in an environment that was humidified and contained 95% air and 5% CO2.

#### Collection of clinical samples

From the First Affiliated Hospital of Dalian Medical University, 6 pancreatic cancer tissues and 6 paired paracancerous tissues were obtained from 2022-01-01 to 2022-05-01. The tissues were derived from surgically resected specimens and rapidly froze in liquid nitrogen to prevent RNA from extracting. All patients signed informed consent forms, which were provided by the First Affiliated Hospital of Dalian Medical University. The paracancerous tissues were situated at a distance of over two centimeters from the neoplasm foci. The standard preoperative care was administered to all of the patients in the research, and neither chemotherapy nor radiation treatment was administered to them. The Ethics Committee of the First Affiliated Hospital of Dalian Medical University granted its approval to conduct this investigation.

#### Isolation of total RNA with quantitative real-time PCR

Using TRIzol (Accurate Biotechnology) as the extraction tool, total RNA was extracted from both primary pancreatic cancer cells and human pancreatic cancer cell lines, as per the guidelines stipulated by the manufacturer. Liquid nitrogen grinding was utilized to extract the RNA from the human pancreatic tissues. The RNA was subsequently subjected to the process of reverse transcription to generate cDNA utilizing an Evo M-MLV RT Kit with gDNA Clean (Accurate Biotechnology). The expression level of 7 ECMGs was measured by RT-PCR utilizing SYBR^®^ Green Premix Pro Taq HS qPCR Kit (Accurate Biotechnology, Shanghai, China), with GAPDH serving as the internal reference. The ΔΔCt method was implemented to analyze and quantify the RNA level expression. The primer sequences employed were as follows, which were obtained from Sangon Biotech (Shanghai) Co., Ltd.: for human COL11A1, 5’- GTGAAGTCATTCAGCCTTTACC-3’(Forward), 5’- ATTTCTTCCATTCCATCCGAGT-3’ (Reverse); for human LAMC2, 5’- GAGGATCAAACAAAAAGCGGAT-3’(Forward), 5’- AGATTCTTCTGTGTACGCTTGA-3’(Reverse); for human LAMA3, 5’- CTGCAGTTTAAACAAACCACCT-3’(Forward), 5’- CAGCTGGTTGATACGAAAAGTC-3’ (Reverse); for human LAMB3, 5’- GAGCCTGTGACTGTGATTTCC-3’(Forward), 5’- GGTAGCGATTACAGTAGCCTC-3’(Reverse); for human ITGA2, 5’- GGGCGACGAAGTGCTACGAAAG-3’(Forward), 5’- ACCCAAGAACTGCTATGCCAAACC-3’ (Reverse); for human ITGB4, 5’- AATGGGGGCATCTGTAATGG-3’(Forward), 5’- GAGTAGTTGATCTCGCAGATGG-3’(Reverse); for human GAPDH, 5’- GGTCTCCTCTGACTTCAACA-3’ (Forward), 5’- GTGAGGGTCTCTCTCTTCCT-3’ (Reverse); for human ITGB6, 5’-ACCTGTGAAGACTGCCTGCTTATTG-3’ (Forward), 5’- ACACCTTTCGCCAACTCCAGATG-3’ (Reverse).

#### Immunohistochemistry, immunofluorescence, and single-cell maps

The protein expression levels of the 7 hub ECMGs were verified between cancer and normal samples from The Human Protein Atlas (HPA: https://www.proteinatlas.org/). However, we did not find the protein expression information of COL11A1 in the HPA database, therefore we displayed the protein expression levels of the other 6 hub ECMGs. In addition, the antibody staining in different types of cancer observed in current human tissue was categorized in the HPA dataset as not observed, low, medium, or high. This score was determined by taking into account both the intensity of staining and the percentage of total cells that were stained. Similarly, the HPA database was also employed to demonstrate the cellular localization (except the COL11A1) through immunofluorescence. Finally, we also analyzed the single-cell types atlas of the 7 hub ECMGs in PAAD tissues with the help of the HPA platform.

### ECM scores and ECMG expression-based cluster analysis

Because of the significant differences in gene expression levels observed in the datasets that had been acquired, we created an ECM-related score classifier premised on the mRNA expression of ECMGs to demonstrate the differential levels of expression present across samples. Specifically, ssGSEA was initially undertaken to assess each patient’s ECM pathway activity. The differential analysis was carried out utilizing “gplots” package in R, and the “pheatmap” package was employed to create the heat map based on the cluster analysis outcomes. The mRNA expression levels in cancerous tissues were categorized by comparisons with the mRNA expression levels of the genes in normal samples: ECM active [cluster 1 (C1)], ECM inactive [cluster 2 (C2)], and normal ECM [cluster 3 (C3)].

We utilized the violin plot that was generated utilizing the ”ggpubr” package to represent the enrichment scores of the clusters to better demonstrate the links that exist between the gene expression levels of these three clusters. The “survival” and “survminer” packages in R were adopted to investigate the discrepancy in prognosis among these three clusters. The “pRRophetic” package in R was utilized to ascertain the half-maximal inhibitory concentration (IC50) of the drugs in the 3 clusters based on the cell expression levels identified from the GDSC(Genomics of Drug Sensitivity in Cancer) database. Of note, the value of the IC50 should be as low as possible for optimal drug sensitivity.

### Effect of differentially expressed tumor suppressor genes and oncogenes in the ECM-receptor interaction

The ECM-receptor interaction performs an instrumental function in modulating the proliferation, differentiation, as well as apoptosis of tumor cells. Aberrant activation of the ECM-receptor interaction was linked to the onset and progression of distinct malignancies. Chromatin modifications, which include ubiquitination, acetylation, and histone methylation, are among the most vital epigenetic processes that modulate gene transcription. Certain classical oncogenes and histone modification-related genes (i.e. sirtuins (SIRTs) and histone deacetylases (HDACs)) in the ECM-receptor interaction might affect the modulation of the ECM-receptor interaction. In our research, the expression levels of several oncogenes were evaluated and displayed using heat maps. These levels were compared across the three clusters of the ECM-receptor interaction.

### Correlation between the ECM scores and tumor immune microenvironment

Quantification of the 29 immune-related gene sets retrieved from TCGA was accomplished with the use of a ssGSEA, and the final number of genes found was 707, reflecting distinct types of immune cells, functions, and pathways (17). After that, a heat map depicting the connection between the ECMGs and infiltration level of immune cells was created with the use of R Studio’s “ggplot2” and “dplyr” packages, utilizing Spearman’s correlation coefficient as an analytical tool for analyzing statistical data. Kruskal-Wallis test was also applied to make a comparison of the discrepancy in the expression of immune checkpoints genes (ICGs) among these three clusters. In a single experiment, the gene signals expression from various immune cell populations may be analyzed utilizing ssGSEA. Depending on the findings of ssGSEA, we utilized the “ggstatsplot”, “data.table”, “dplyr”, “tidyr”, and “ggplot2” packages in RStudio to evaluate and visualize the association between the ECM score and immune substances. In the plotted figure, the degree of association is represented by the area of each sphere, and the p-value is represented by the color. Lastly, we utilized the “ggscatterstats” package to construct a scatter plot for displaying the associations between the 4 classical immune cell populations (T helper (Th) cells, regulatory T cells (Treg), neutrophils, and macrophages) and the ECM score.

### Establishment and verification of a novel ECM-associated prognostic panel for PAAD patients

To clearly estimated the survival probability of PAAD patients, a novel prognostic panel was constructed and validated premised on the expression of ECM-associated mRNAs and long non-coding RNAs (lncRNAs). After retrieving the human gene transfer format (gtf) file from the Ensembl database (http://www.ensembl.org/), it was used to differentiate between protein-coding genes and lncRNAs. The ‘cor.test’ built-in function in R was employed to determine the Pearson correlation coefficients between ECM-associated mRNAs and all lncRNAs. After that, the lncRNAs that had |correlation coefficients| >0.4 and p values <0.001 were taken into consideration as ECM-associated candidates for subsequent analyses. Afterward, the expression levels of ECM-associated lncRNAs and mRNAs were integrated with clinical data from the TCGA dataset and the ICGC dataset, correspondingly. To determine whether ECM-associated mRNAs and lncRNAs have prognostic significance, a univariate Cox regression analysis was executed (filter criteria: p<0.05).

The 177 samples included in the TCGA dataset were categorized at random into two groups: the train cohort, which consisted of 89 individuals, and the test1 cohort, which consisted of 88 individuals. Furthermore, all of the samples from the TCGA dataset were allotted to the test2 cohort (n=177), and all of the samples from the ICGC cohort were allotted to the test3 cohort (n= 82).

To prevent over-fitting and identify suitable variables among the ECM-associated mRNAs and lncRNAs with prognostic significance, a least absolute shrinkage and selection operator (LASSO) regression analysis was conducted on the train cohort. After that, a multivariate Cox proportional hazards regression analysis was employed to establish an ECM-APP, and the “predict” function in R was utilized to calculate the risk score. Following the completion of the calculation of each sample’s risk score in the train cohort, 89 samples were separated into low- and high-risk populations premised on the median risk score. The PAAD patients who enrolled in the test1, test2, and test3 cohorts were subsequently categorized into low- and high-risk populations premised on the median risk score that was acquired in the train cohort.

The following investigations were carried out on the train, test1, test2, and test3 cohorts with the aim of conducting both internal and external validation of the ECM-APP (1): The ‘pheatmap’ R package was used to generate a heatmap that depicts the varying levels of gene expression that are associated with ECM-APP (2). The Kaplan-Meier method was utilized to carry out a survival analysis to determine whether or not the ECM-APP can accurately predict survival (3); The area under the curve (AUC) served as the basis for our receiver-operating characteristic (ROC) curves, which we utilized to determine the diagnostic significance of the ECM-APP (4); The ECM-APP was evaluated utilizing univariate and multivariate Cox regression analyses, correspondingly, to identify whether or not it had independent prognostic value.

### Tumor mutation burden analysis

The aggregate number of somatic gene insertion errors, deletion errors, gene coding errors, and base substitutions identified per million bases were described as the TMB. The “perl” programming language was utilized to count the number of non-synonymous mutations. Then, the differences in TMB across the high- and low-risk populations were computed with P <0.05 signifying the criteria for statistical significance. The PAAD driver genes were then identified utilizing the R package “maftools,” and the state of the topmost 20 genes exhibiting the greatest mutation frequency in the low- and high-risk populations was investigated further. We also explored the discrepancy in the clinical outcomes between different TMB scores and ECM-APP risk scores through K-M log-rank test.

### Analysis of the tumor immune microenvironment in low - and high-risk population

To get a deeper comprehension of the possible processes behind the different prognoses across high- and low-risk subgroups, we intensively studied the discrepancies in immunocyte infiltration, immune function response, and immune checkpoint expression based on a variety of bioinformatics algorithms.

The TIMER2.0 database (http://timer.cistrome.org/) offers a complete immunological signature of tumor-infiltrating cells in a wide variety of tumor samples taken from the TCGA database. This signature is premised on the algorithms of QUANTISEQ, MCPCOUNTER, EPIC, XCELL, CIBERSORT, and TIMER. The “pheatmap” package in R was used to illustrate the infiltration levels of a variety of immune cells within each sample belonging to the train, test1, and test2 cohorts. After that, the “limma” package in R was adopted to determine the statistical differences in infiltration levels of immune cells between low- and high-risk subgroups. The heatmap revealed only immune cells that had significant variation (p < 0.05) In addition, the “gsva” package in R was utilized in conducting ssGSEA to discover the infiltration levels of immunocytes and immune-associated functions that were different between the high-risk and low-risk subgroups. Ultimately, only the statistically significant findings were displayed after investigating the differential expression of common ICGs in high- and low-risk subgroups (filter criteria: p < 0.05).

## Results

### Pan-cancer landscape of ECMGs

**Figure 1:**
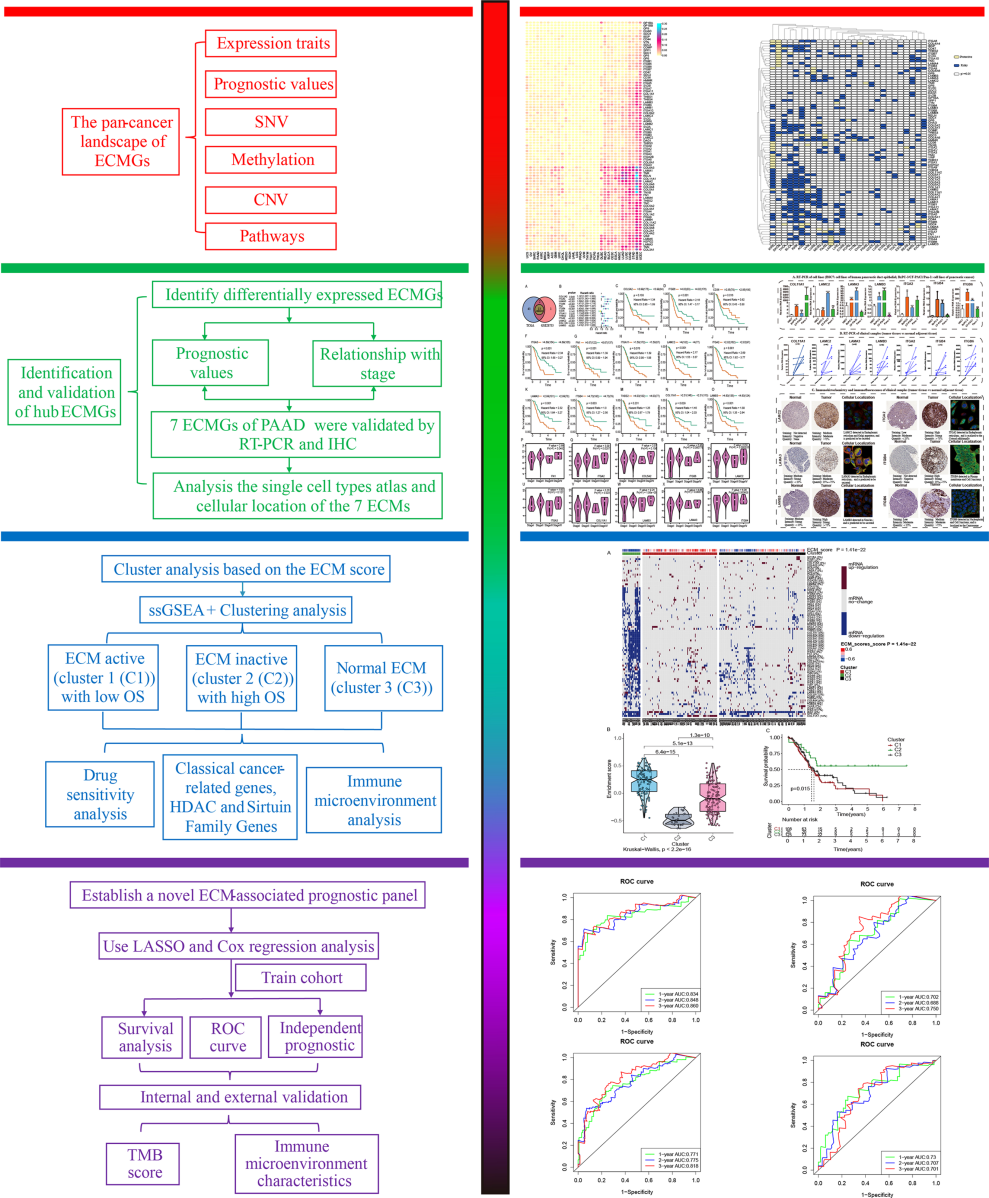
The current study’s flowchart.

**Figure 2:**
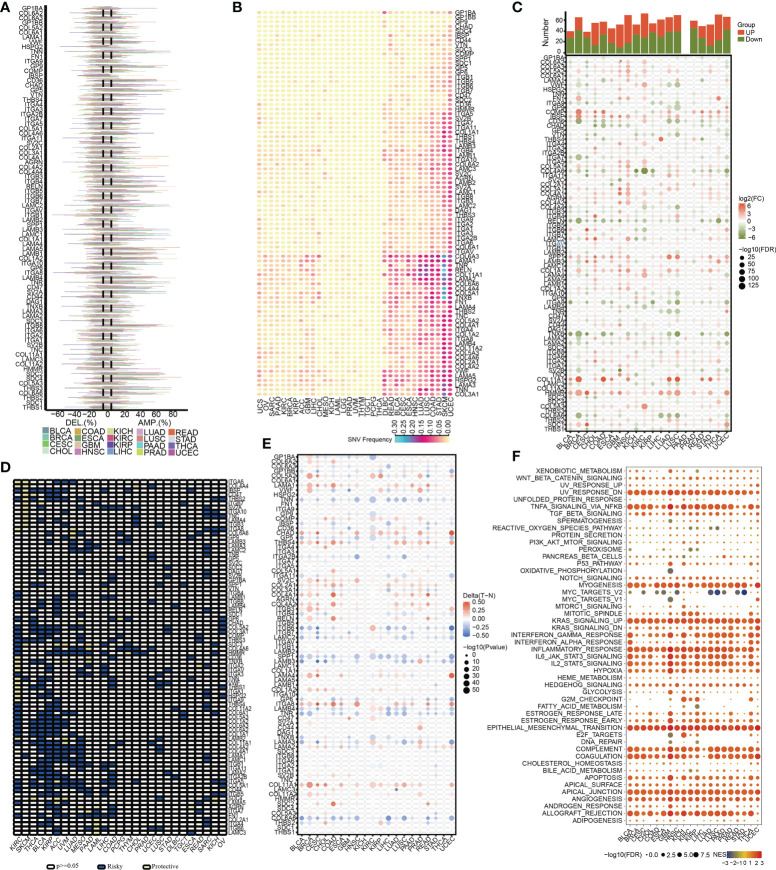
The pan-cancer landscape of ECM receptor interaction-related genes. **(A)** CNV gain and loss frequency data across cancer types. The length of the line represents the variation frequency of the ECM-related genes in pan-cancers. **(B)** SNV data across cancer types. The mutant frequencies of ECM-related genes in pan-cancers (red color to green color represents low to high). **(C)** Gene expressions of the ECM-associated genes between tumor and neighboring normal tissues in pan-cancers (P<0.05). **(D)** Survival landscape of the ECM-related genes among all the types of cancers. The white color represents genes with P > 0.05, and the blue and yellow colors represent risk and protective genes, respectively. **(E)** The DNA methylation of ECMGs in different tumors (red color to blue color signifies high to low). **(F)** The relationship between cancer signaling and ECMGs (red color to blue color signifies high to low).

To begin, we created a corresponding flowchart (**Figure 1**) to depict the workflow of this study project more vividly. To examine the genetic abnormalities of ECMGs in cancer, the proportion of CNV was analyzed, and the findings revealed that in general CNV occurred at high frequencies (almost ranging from 10% to 40%) in most cancer types (**Figure 2A**). We discovered that UCEC, SKCM, STAD, COAD, and LUSC, as well as LUAD, all had a greater frequency of SNVs. On the other hand, the frequency of SNVs found in UCS, OV, SARC, PAAD, and KIRC was low (**Figure 2B**).

To examine the mRNA expression status of ECMGs between tumor and neighboring normal samples in various cancers, differential expression analysis was performed (**Figure 2C**). The levels of COL5A2, IBSP, COL4A1, AGRN, SPP1, COL11A1, and HMMR in nearly all tumor tissues were significantly elevated compared with those in paired normal tissues; on the contrary, the levels of RELN, ITGA8, TNXB, LAMA2, SV2B, and COL6A6 in nearly all tumor tissues were significantly decreased compared with those in paired normal tissues. Subsequently, we then intensively investigated the prognostic performances of these ECMGs in pan-cancers by analyzing gene expression and patients’ survival time. As illustrated in **Figure 2D**, we executed a univariate cox regression analysis to discover the risky ECMGs (HR>1 and p<0.05) and protective ECMGs (HR<1 and p<0.05). Interestingly, we found that most ECMGs are present as risk factors in most tumors including PAAD; however, part of ECMGs function as protective factors in KIRC and SKCM.

In addition to CNV, methylation of promoters may influence gene expression, and abnormal DNA methylation of promoters is linked to the onset of tumors. In each of the 20 different types of cancer, we found that ECMGs had complicated methylation patterns. LAMA1, CHAD, ITGA8, and COL11A1 consistently showed hypermethylation in more than half of cancer types; on the contrary, TNN, SPP1, COL6A6, and TNR consistently showed hypomethylation in more than half of types of cancer (**Figure 2E**). Additionally, the links between cancer hallmarks and ECMGs were evaluated, and the findings revealed that more than half of the hallmarks were frequently remarkably linked to ECMGs (**Figure 2F**).

### Identification and validation of hub ECMGs linked to the onset and progression of PAAD

**Figure 3:**
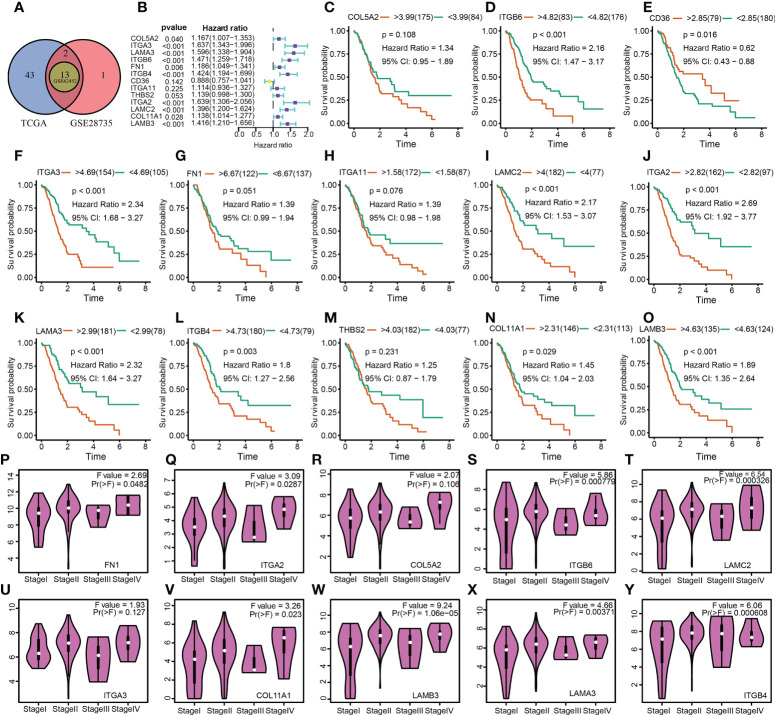
Determination of differentially expressed ECM-related genes, their prognostic significance, and their relationship with the tumor stage of PAAD. **(A)** A Venn diagram revealed 13 ECM-related genes that were differently expressed between PAAD and normal pancreatic tissues. **(B)** The analysis illustrated the prognostic significance of 13 differentially expressed ECM-related genes in PAAD. **(C–O)** Survival analysis of 13 ECM-related genes. **(P–Y)** The relationship between 10 ECM-related genes and the tumor stage of PAAD.

A total of 13 differentially expressed ECMGs between PAAD samples and adjacent/normal pancreas tissues were identified after the intersection of three cohorts’ results (i.e. GSE28735, GSE62452, and TCGA-GTEx) (**Figure 3A**). Consequently, the prognostic values of 13 differentially expressed ECMGs in PAAD were explored by combining Kaplan–Meier survival analysis and univariate Cox regression analysis. As depicted in **Figure 3B**, the findings of univariate Cox regression analysis illustrated that 10 genes (i.e. COL5A2, ITGA3, LAMA3, ITGB6, FN1, ITGB4, ITGA2, LAMC2, COL11A1, and LAMB3) of 13 differentially expressed ECMGs were detected to be significantly associated with PAAD prognosis. Meanwhile, Kaplan–Meier survival analysis demonstrated that 9 genes (i.e. ITGB6, CD36, ITGA3, LAMC2, ITGA2, LAMA3, ITGB4, COL11A1, and LAMB3) of 13 differentially expressed ECMGs were discovered to be remarkably linked to PAAD prognosis (**Figures 3C–O**). Based on the intersection of results of univariate and Kaplan–Meier survival analyses, we obtained 8 ECMGs truly associated with PAAD prognosis (i.e. ITGB6, ITGA3, LAMC2, ITGA2, LAMA3, ITGB4, COL11A1, and LAMB3). Furthermore, we evaluated the possible link between the ECMGs and the tumor stage of PAAD, and 8 ECMGs (i.e. FN1, LAMA3, ITGB6, ITGB4, ITGA2, LAMC2, COL11A1, LAMB3) were detected to be significantly associated with tumor stage (**Figures 3P–Y**). Overall, we identified 7 hub ECMGs (i.e., LAMA3, ITGB6, ITGB4, ITGA2, LAMC2, COL11A1, LAMB3), which were not only differentially expressed in PAAD and adjoining/normal pancreas tissues but also remarkably linked to the prognosis and stage of PAAD.

**Figure 4:**
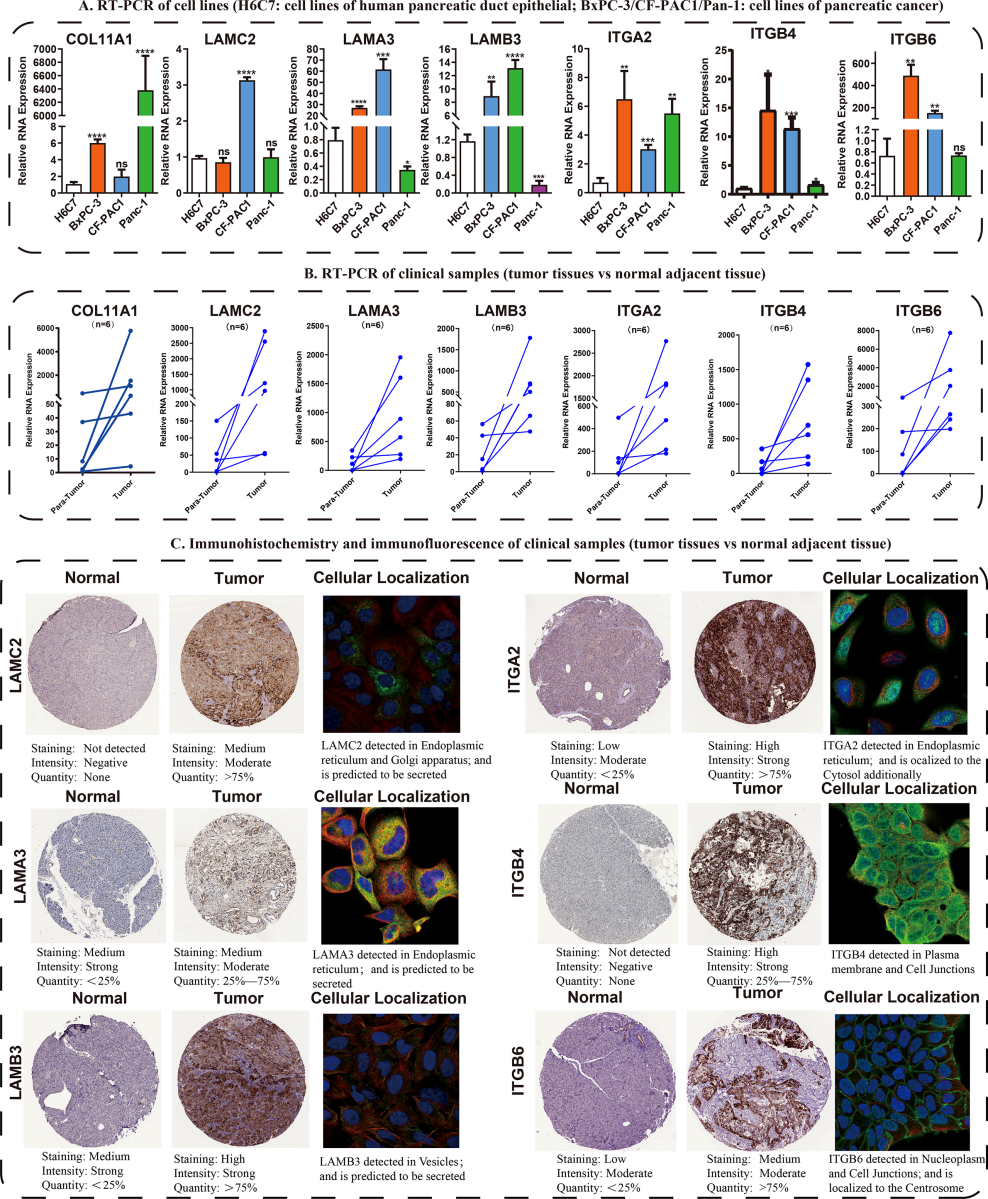
Validation of the expression traits of seven hub ECMGs. **(A)** RT-PCR of cell lines (H6C7: cell lines of human pancreatic duct epithelial; BxPC-3/CF-PAC1/Pan-1: cell lines of pancreatic cancer). **(B)** RT-PCR of clinical samples (tumor tissues vs normal adjacent tissue). **(C)** Immunohistochemistry and immunofluorescence of clinical samples (tumor tissues vs normal adjacent tissue). (* indicates p <0.05; ** indicates p < 0.01; *** indicates p < 0.001; **** indicates p < 0.0001; ns, non-significant

Following these, the expression profiles of 7 hub ECMGs were validated by RT-PCR and IHC in cell lines and clinical samples of PAAD (**Figures 4A–C**). We found the mRNA levels of LAMA3, ITGB6, ITGB4, ITGA2, LAMC2, COL11A1, and LAMB3 were higher in at least one of the pancreatic cell lines than that in normal cell lines cells (**Figure 4A**). Meanwhile, we also explored the expression levels of the 7 hub ECMGs in our clinical samples, and the results showed that the 7 hub ECMGs were expressed at high levels in tumors in contrast with the adjoining non-tumor tissues (**Figure 4B**). To analyze the protein expression of 7 hubs ECMGs, we intensively studied the immunohistochemistry findings of tumor and normal pancreas tissues using the HPA database (**Figure 4C**). We did not find the protein expression information of COL11A1 in the HPA database, therefore, the other 6 hub ECMGs proteins available were performed, which is nearly consistent with the results of the bioinformatic analysis and qRT-PCR. These protein expression levels became markedly up-modulated in PAAD as opposed to normal pancreas tissues. Similarly, we explored the cellular localization of the 6 hub ECMGs (except the COL11A1), the expression product of LAMC2, LAMA3, LAMB3, ITGA2, ITGB4, and ITGB6 were mainly located on the endoplasmic reticulum and Golgi apparatus, endoplasmic reticulum, vesicles, nucleoplasm, plasma membrane, and cell Junctions, and cell Junctions, respectively (**Figure 4C**). By the HPA database, we analyze the single-cell types atlas for each of the 7 hub ECMGs (**Supplementary Figure S1**). We found that COL11A1 is mainly distributed in pancreatic smooth muscle cells, while other genes are mainly distributed in pancreatic endocrine cells.

### Cluster analysis premised on the ECM scores

**Figure 5:**
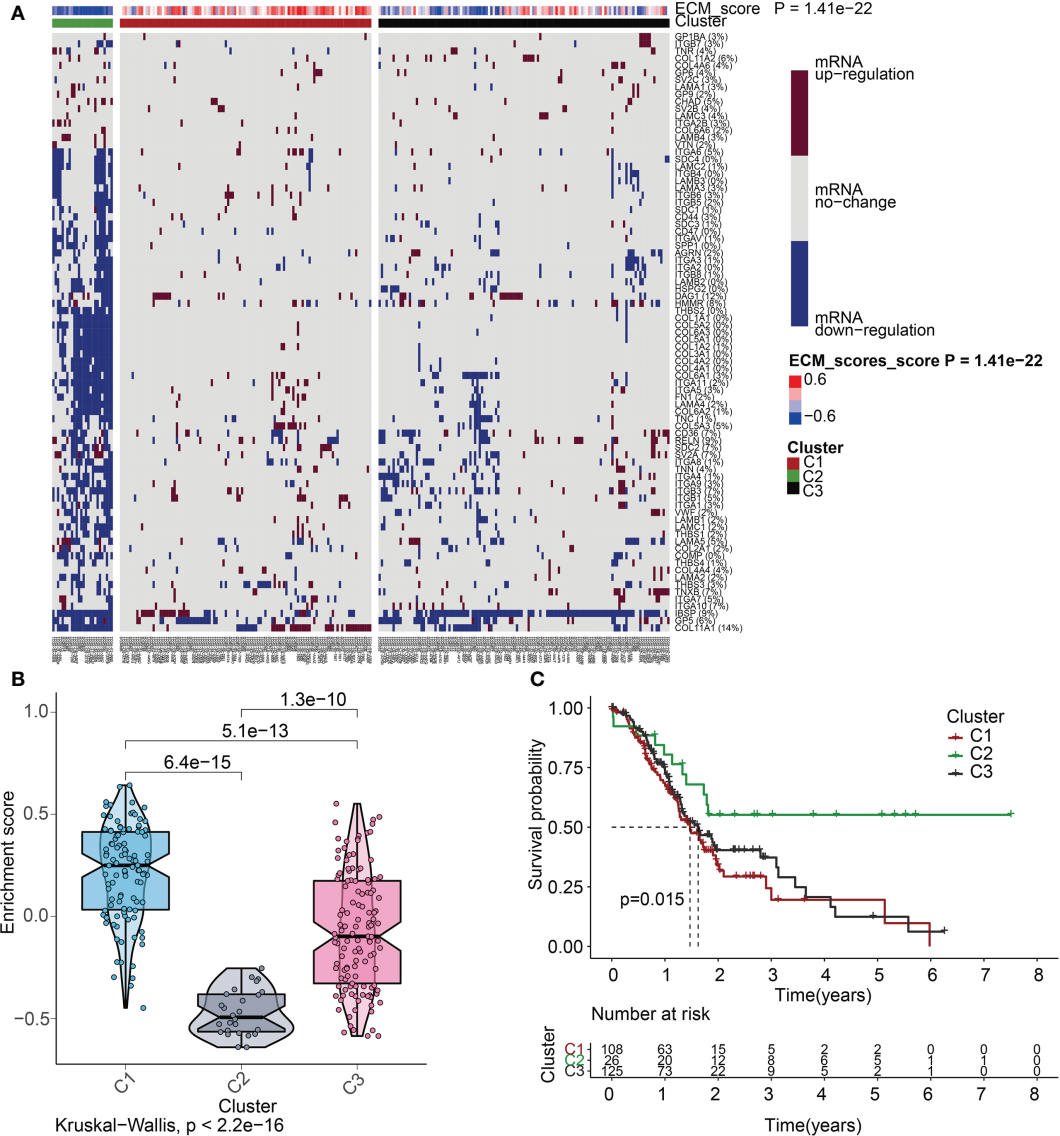
ECM score-based clustering analysis. **(A)** Clustering of gene data illustrates 3 clusters displayed by the heat map: ECM active [cluster 1 (C1)], ECM inactive [cluster 2 (C2)], and normal ECM [cluster 3 (C3)]. The proportion of patients with upmodulated genes is shown on the right side of the figure. In the color bar on the right side, red denotes mRNA up-modulation, blue signifies mRNA downmodulation, and white denotes mRNA no-regulation. The ECM score is displayed in four different colors, with negative values shown in blue and positive ones shown in red. **(B)** The “ggpubr” package’s violin plot indicates that the enrichment scores of the three clusters, from high to low, are C2, C3, and C1. The “kruskal.test” was employed in this study, and the p-values are presented above the clusters. **(C)** A survival curve depicting the three different clusters. Cluster 2 has a better survival rate compared to either cluster 1 or cluster 3. Cluster 1 is denoted by the red line, cluster 2 is denoted by the green line, and cluster 3 is denoted by the black line. The number of years is signified by the abscissa, while the rate of survival is denoted by the ordinate.

To delve more into the link between ECMGs and PAAD, we classified 259 samples of PAAD into three distinct groups depending on the levels of mRNA expression of ECMGs (**Figure 5A**). C1 contained tumor patients with active ECM, C2 contained those with inactive ECM, and C3 contained those with normal ECM. The enrichment scores for the 3 clusters were orderly arranged as C1 > C3 > C2 according to the violin plot (**Figure 5B**). Then, we assessed whether or not the clustering was plausible by generating the survival curves for each of the 3 clusters. Patients diagnosed with PAAD who were allocated to the C2 cohort exhibited remarkably higher OS rates as opposed to those allocated to the C1 and C3 clusters (**Figure 5C**), suggesting that the ECM score was an indicator that should be considered risky.

### Link between drug sensitivity and ECM clusters

**Figure 6:**
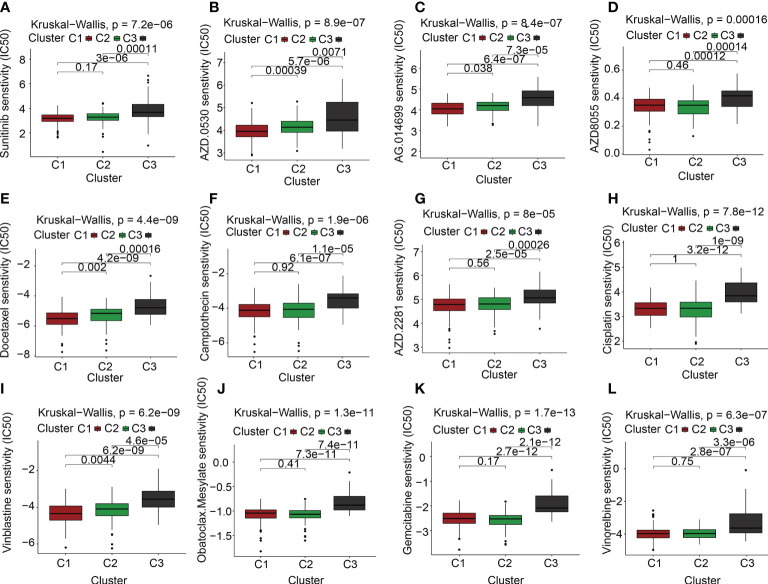
The link between ECM clusters and drug sensitivity. The estimated IC50 box plots for 12 kinds of commonly used chemotherapeutic drugs are conducted in **(A-L)** for cluster 1 (yellow), cluster 2 (blue), and cluster 3 (red). The 12 types of chemotherapeutic agents are AZD0530 (saracatinib), sunitinib, AZD8055 (mTOR inhibitor), AG.014699 (rucaparib), AZD.2281(olaparib), camptothecin, docetaxel, cisplatin, vinblastine, vinorelbine, gemcitabine, obatoclax.Mesylate. The P-values are depicted above the clusters and the P < 0.05 is deemed as having statistical significance.

Targeted molecular medicines are now the primary treatment option for individuals with advanced PAAD who have lost the chance to undergo surgical resection. The molecular biology of cancer serves as the foundation for molecular targeted treatment, which treats cancer by setting tumor-associated molecules as therapeutic targets and using medications and agents that are highly specific to those molecules. As described previously, the ECM is a significant contributor to the onset and progression of malignancies. Our findings also suggested that the ECMGs might have a risky function in PAAD. Is there any connection between the ECMGs and the currently available targeted medications that can efficiently treat PAAD? To obtain greater insights into this topic, we conducted further analysis on the theoretical grounds and instructions provided for PAAD to investigate the connection between ECMGs and the IC50 values of 12 targeted treatments and traditional drugs that are often used in tumor research [35,36]. The 12 drugs we selected included the use of tyrosine kinase inhibitor(e.g., saracatinib, sunitinib), mTOR inhibitor, poly ADP-ribose polymerase inhibitor(e.g., rucaparib, olaparib), plant anticancer drugs(e.g., camptothecin, docetaxel, cisplatin, vinblastine, vinorelbine), the first-line chemotherapeutic drugs, pyrimidine antineoplastic drugs(e.g., gemcitabine), and Bcl-2 inhibitor(e.g., obatoclax. Mesylate). With the help of the “pRRophetic” package, we predicted the IC50 values of above 12 commonly targeted drugs among these three molecular subtypes. A lower value for IC50 is an indication of increased sensitivity to the medication. Following is a list of the various medication sensitivities that were recorded among the ECM clusters based on the research results: AZD0530(saracatinib): C1 > C2 > C3; sunitinib: C2 > C3; AZD8055(mTOR inhibitor): C2 > C1 > C3; AG.014699(rucaparib): C1 > C2 > C3; AZD.2281(olaparib): C2 > C3; camptothecin: C2 > C3; docetaxel: C1 > C2 > C3; cisplatin: C2 > C3; vinblastine: C1 > C2 > C3; vinorelbine: C2 > C3; gemcitabine: C2 > C1 > C3; obatoclax.Mesylate: C2 > C3 (**Figures 6A–L**).

### Correlation of ECM scores with potentially targetable classical genes, HDAC family genes, and sirtuin family genes

**Figure 7:**
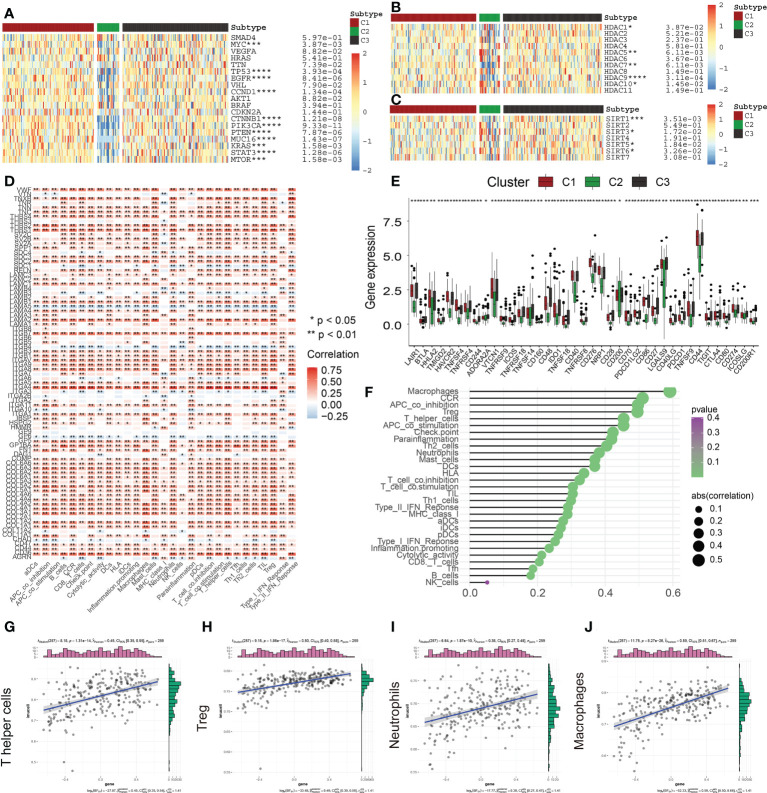
Correlation of ECM scores with Classical Oncogenes, Tumor Suppressor Genes, and Immune Cell Infiltration. **(A)** Different levels of expression of distinct tumor suppressor genes and oncogenes in 3 ECM clusters. **(B)** The different levels of HDAC Family Genes expression among three ECM clusters. **(C)** The different levels of Sirtuin Family Genes expression across the 3 ECM clusters. **(D)** The heat map depicts the relationship between ECM-associated genes and infiltration levels of immune cells. A positive association is denoted by red, while a negative association is denoted by blue. **(E)** The different expression levels of immune checkpoints among three ECM clusters. **(F)** The plot depicts the link between the ECM score and infiltration levels of immune cells. The size of the sphere on the right side of the figure denotes the degree of abs (correlation), while the color denotes the p-value. **(G–J)** The scatter plot depicts the relationship of ECM score with 4 immune-infiltration-related substances. The ECM score was shown to have a positive link to the infiltration levels of neutrophils, macrophages, CCR, Treg cells, and so on. (* indicates p <0.05; ** indicates p < 0.01; *** indicates p < 0.001; **** indicates p < 0.0001).

Heat maps relating to oncogenes in the three ECM clusters were produced to subsequently examine the differential expression profiles of the oncogenes in each of the clusters (**Figure 7A**). Interestingly, we discovered that the levels of expression of the oncogenes MTOR, KRAS, MYC, CCND1, and PIK3CA were much lower in C2 in comparison to C1 and C3. The inhibition of oncogenes probably accounts for the better prognosis of C2 subgroups. In addition, the expression levels of commom tumor suppressor genes including TP53 and PTEN were lowered in C2 but elevated in C1 and C3, similar to the expression levels of the oncogenes.

Gene transcription mediation is greatly influenced by the activity of enzymes known as histone deacetylases. These enzymes are responsible for catalyzing the elimination of acetyl groups from histone and non-histone lysine residues and are intimately linked to tumor onset and metastasis (18). Recently, suppression of HDAC has emerged as a method that has been verified in clinical practice for treating cancer (19). The levels of expression of HDAC1, HDAC7, and HDAC9 were found to be considerably lowered in C2 contrasted with the levels observed in C1 (**Figure 7B**). In addition, the levels of HDAC5 and HDAC10 expression were remarkably elevated in C2 (**Figure 7B**). These findings revealed that various members of the HDAC family had varied functions in the PAAD and may provide novel avenues for the future targeted therapy of malignancies.

Histone deacetylation enzymes (HDACs) of classes I and II are not the only types of HDACs and there is another particular class of HDACs (class III HDAC, Sirtuin) (20). Recently, a vast growing body of research indicates that SIRTs engaged in a wide variety of biological activities that are relevant to the onset and advancement of tumors, including variations in both the tumor-associated metabolic pathways and TME and uncontrolled proliferation of cells (21, 22). SIRTs are considered to perform a complicated function as oncogenes or tumor inhibitors in a variety of cancers under a variety of experimental settings. In this research, SIRT3 and SIRT6 were highly expressed in the C2 subgroup; however, SIRT1 and SIRT5 had low expression in the C2 subgroup (**Figure 7C**). Accumulating evidence has suggested that SIRT3 and SIRT6 could be served as the tumor suppressors, thus, the abnormal sirtuin status might be also responsible for the different clinical outcomes among three ECM-based clusters (22).

### Correlations between the ECM scores and the immune cell infiltration

The TME is a dynamic environment that involves two-way interaction between stromal and tumor cells and is characterized by the presence of complex networks (23, 24). During the process of tumor cell development, immune evasion takes place, which later develops into metastases, and the ECM offers the molecular and cellular milieu that is essential for this dynamic mechanism. Immune cells, which are an essential component of the TME, are intimately linked to the clinical manifestation of neoplasms and hence have the potential to serve as effective therapeutic targets for cancer (25). Furthermore, the ECM is very crucial in the process of modulating the immunological microenvironment of tumors (26, 27). In this research, we further examined the link between ECMGs and infiltration levels of immune cells and immune-associated roles (**Figure 7D**). As per the findings, the vast majority of genes have a positive association with the vast majority of immune-infiltrating agents, including ITGA4. In contrast, some genes, such as LAMA5, ITGB4, GP6, AGRN, ITGA3, and SDC1, are inversely linked to the infiltration levels of immune cells. It has been well-established that ICGs have an instrumental function in modulating the functionality of effector T cells and, as a consequence, anti-tumor immunity. Our findings reveal that ECM-inactive subgroups are accompanied by a lower expression level of ICGs, indicating ECM-inactive subgroups had a stronger anti-tumor immune response and a better prognosis (**Figure 7E**). Following these, we additionally examined whether or not there was a connection between the ECM score and infiltration levels of immune cells (**Figures 7F–J**). Consequently, it was shown to have a favorable link to the infiltration levels of most immune cells, including Th cells, Treg cells, neutrophils, and macrophages.

### ECM-associated prognostic panel enables the auxiliary prediction of clinical outcomes of patients with PAAD

In addition to well-recognized ECM-related mRNAs, multiple lncRNAs also had the potential to react with ECM and then influence proliferation, apoptosis, metastasis, and invasion of various tumor cells. To construct a comprehensive and robust ECM-APP, we first identify 278 ECM-related lncRNAs with the help of the Pearson correlation test (**Supplementary Figure S2A**). Subsequently, we integrated the expression profiles of 83 ECM-related mRNAs and 278 ECM-related lncRNAs and rated them as candidate molecules used for the panel development. As per the findings of a univariate Cox regression analysis, 149 out of 361 candidate molecules served as possible prognostic markers (**Supplementary Figure S2B**). After this, a LASSO regression analysis was carried out to get rid of any collinearity that existed across the 149 genes and to avoid the overfitting of the prognostic model (**Supplementary Figure S2C**). 13 of 149 candidate molecules were preserved for additional multivariate Cox regression analysis (**Supplementary Figure S2D**). Lastly, multivariate Cox proportional hazards regression analysis integrating nine genes (i.e., PTOV1−AS2, RPARP−AS1, CASC8, UNC5B−AS1, ITGA7, AC136475.3, AC027288.3, LINC01116, UCA1) was performed to establish a comprehensive and robust ECM-APP (**Supplementary Figure S2E**).

**Figure 8:**
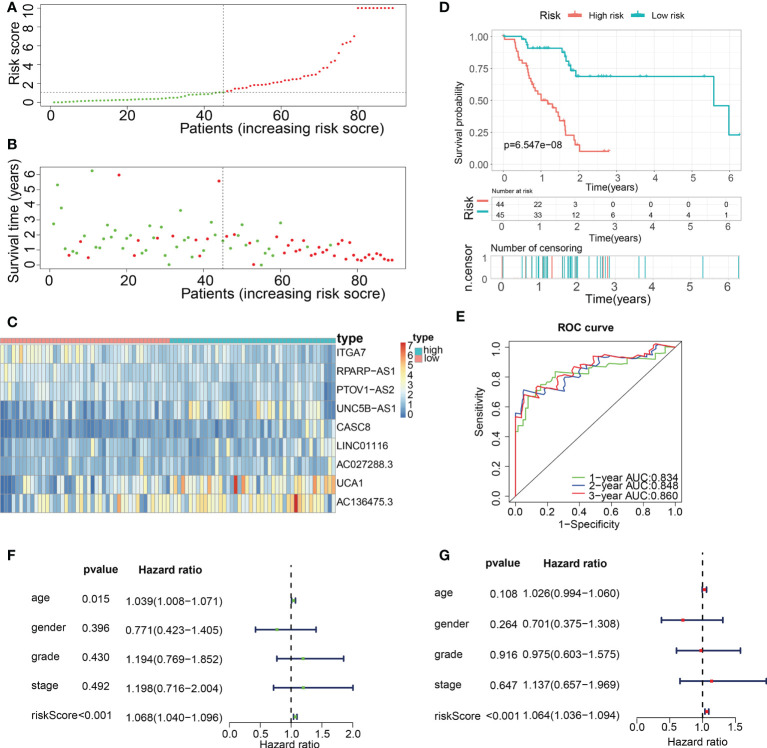
Construction of a novel ECM-APP in the train cohort. **(A)** Sectionalization premised on the median risk score. **(B)** Survival status and risk score distributions **(C)** Heatmap depicted the expression levels of 9 genes integrated into the prognostic signature. **(D)** Survival curve of OS time in low-risk and high-risk subgroups. **(E)** ROC curve of 1-, 2-, and 3-years of train cohort, area under the curve (AUC) of the curve are 0.834, 0.848, and 0.860, correspondingly. **(F)** Univariate cox regression analysis illustrated the independence of risk score, gender, grade, stage, and age. **(G)** Multivariate cox regression analysis showed the independence of risk score, stage, grade, gender, and age.

Subsequently, in the train cohort, we separated 89 PAAD patients into low- and high-risk subgroups premised on the median risk score of 1.092 (**Figure 8A**). Predicated on the risk score distributions and the survival status, the high-risk subgroup of PAAD patients had a significantly higher death rate (**Figure 8B**). The heatmap depicted the expression distributions of 9 ECMGs involved in the prognostic panel (**Figure 8C**). Then, the findings of the Kaplan-Meier survival analysis highlighted that PAAD patients had a worse OS in the high-risk subgroup in contrast with those in the low-risk subgroup, implying that the ECM-APP could accurately distinguish PAAD patients with poor or good prognosis (**Figure 8D**). To confirm the effectiveness and accuracy of ECM-APP, the diagnostic performance of the risk score was evaluated utilizing the ROC analysis. The AUCs of the ROC curves had values of 0.834, 0.848, and 0.860, correspondingly, for 1-, 3-, and 5- year survival (**Figure 8E**). Considering that the c-index for the train set was 0.847, it is reasonable to infer that the ECM-APP had an outstanding prognostic ability. Finally, using the clinical follow-up data acquired from TCGA, we conducted univariate and multivariate Cox regression analyses. The findings illustrated that the risk score independently functioned as a prognostic index (HR = 1.068, 95% CI =1.040−1.096, p < 0.001 in univariate analysis; HR =1.064, 95%CI = 1.036−1.094, p < 0.001 in multivariate analysis) (**Figures 8F, G**). It is worth noting that stage, grade, gender, and age, were not the independent prognostic markers for PAAD patients, which illustrated the integrity and accuracy of our model.

**Figure 9:**
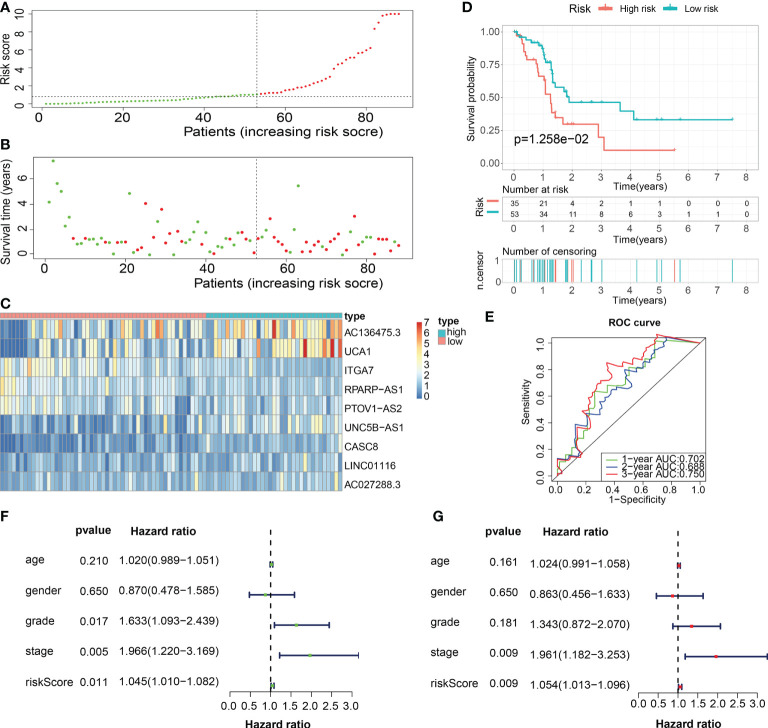
Internal validation of the robust ECM-APP in the test1 cohort. **(A)** Sectionalization premised on the median risk score. **(B)** Survival status and risk score distributions **(C)** Heatmap depicted the expression profiles of 9 genes integrated into the prognostic signature. **(D)** Survival curve of OS time in low-risk and high-risk subgroups. **(E)** ROC curve of 1-, 2-, and 3-years of test1 cohort, area under the curve (AUC) of the curve are 0.702, 0.688, and 0.750, correspondingly. **(F)** Univariate cox regression analysis illustrated the independence of risk score, stage, grade, gender, and age. **(G)** Multivariate cox regression analysis showed the independence of risk score, stage, grade, gender, and age.

**Figure 10:**
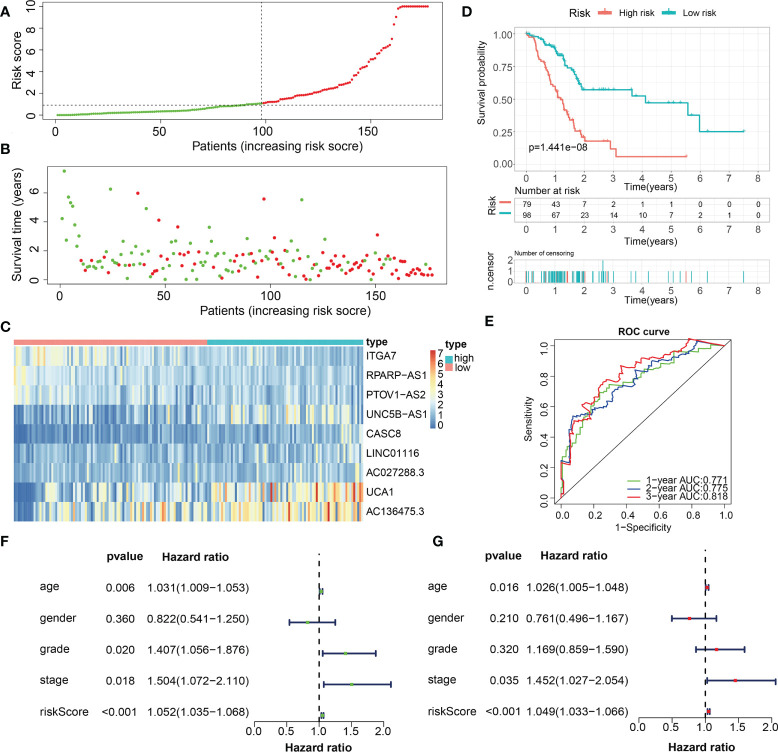
Internal validation of the robust ECM-APP in the test2 cohort. **(A)** Sectionalization premised on the median risk score. **(B)** Survival status and risk score distributions. **(C)** Heatmap illustrated the expression profiles of 9 genes integrated into the prognostic signature. **(D)** Survival curve of OS time in low-risk and high-risk subgroups. **(E)** ROC curve of 1-, 2-, and 3-years of in test2 cohort, area under the curve (AUC) of the curve are 0.771, 0.775, and 0.818, correspondingly. **(F)** Univariate cox regression analysis showed the independence of risk score, stage, grade, gender, and age. **(G)** Multivariate cox regression analysis showed the independence of risk score, stage, grade, gender, and age.

**Figure 11:**
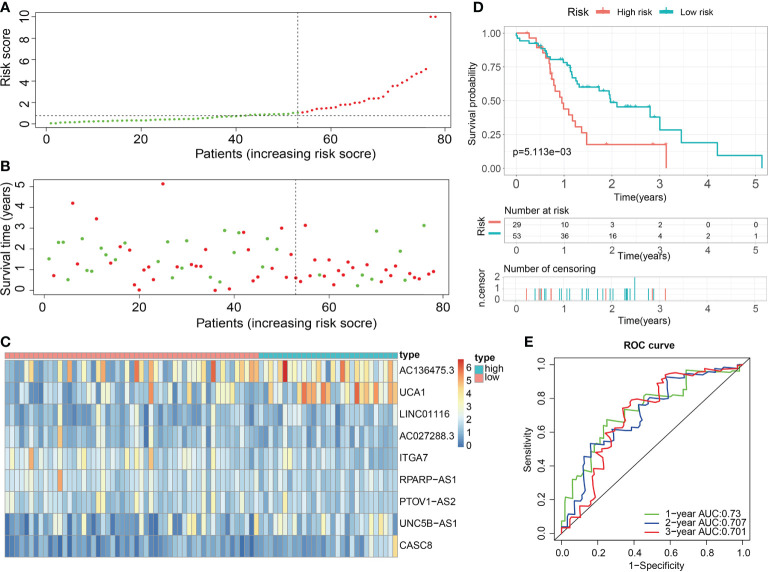
External validation of the robust ECM-APP in the test3 cohort. **(A)** Sectionalization premised on the median risk score. **(B)** Survival status and risk score distributions. **(C)** Heatmap demonstrated the expression profiles of 9 genes integrated into the prognostic signature. **(D)** Survival curve of OS time in low-risk and high-risk subgroups. **(E)** ROC curve of 1-, 2-, and 3-years of in test3 cohort; areas under the curve (AUC) of the curve are 0.73, 0.707, and 0.701, correspondingly.

For validation of the reliability of the ECM-APP, PAAD samples in the test1 cohort, test2 cohort, and test3 cohort were also distinguished into low- and high-risk populations with a similar method (**Figures 9A**, **10A**, **11A**). Notably, when differentiating across the samples, the median risk score of 1.092 in the train cohort functioned as a unified benchmark. The risk score distributions, as well as survival status in the three cohorts, revealed similar tendencies to those reported in the train cohort in both the internal validation cohorts (test1 and test2 cohorts) and the external validation cohort (test3 cohort) (**Figures 9B**, **10B**, **11B**). Additionally, the heatmaps from the test1, test2, and test3 cohorts exhibited a similar expression pattern of the 9 ECMGs to that in the train cohort (**Figures 9C**, **10C**, **11C**). More importantly, Both the internal and the external validation results showed that patients diagnosed with PAAD who had high risk scores exhibited dismal OS rates (all p < 0.05) (**Figures 9D**, **10D**, **11D**). We discovered that in the test1 cohort, the AUC values of the ROC curves were 0.702, 0.688, and 0.705 while in the test2 cohort, they were 0.771, 0.775, and 0.818 for 1-, 3-, and 5-year survival, correspondingly. Moreover, the AUC values in the external validation were 0.730, 0.707, and 0.701 in the test3 dataset (**Figures 9E**, **10E**, **11E**). In the test1, test2, and test3 datasets, the c-index was computed to be 0.698, 0.788, and 0.713, correspondingly. Lastly, due to the deficiency of clinical information in ICGC, our novel ECM-APP was also proved to be an independent prognostic indicator in test1and test2 cohorts (**Figures 9F, G**, **10F, G**).

### Characterization of TMB between low- and high-risk populations

**Figure 12:**
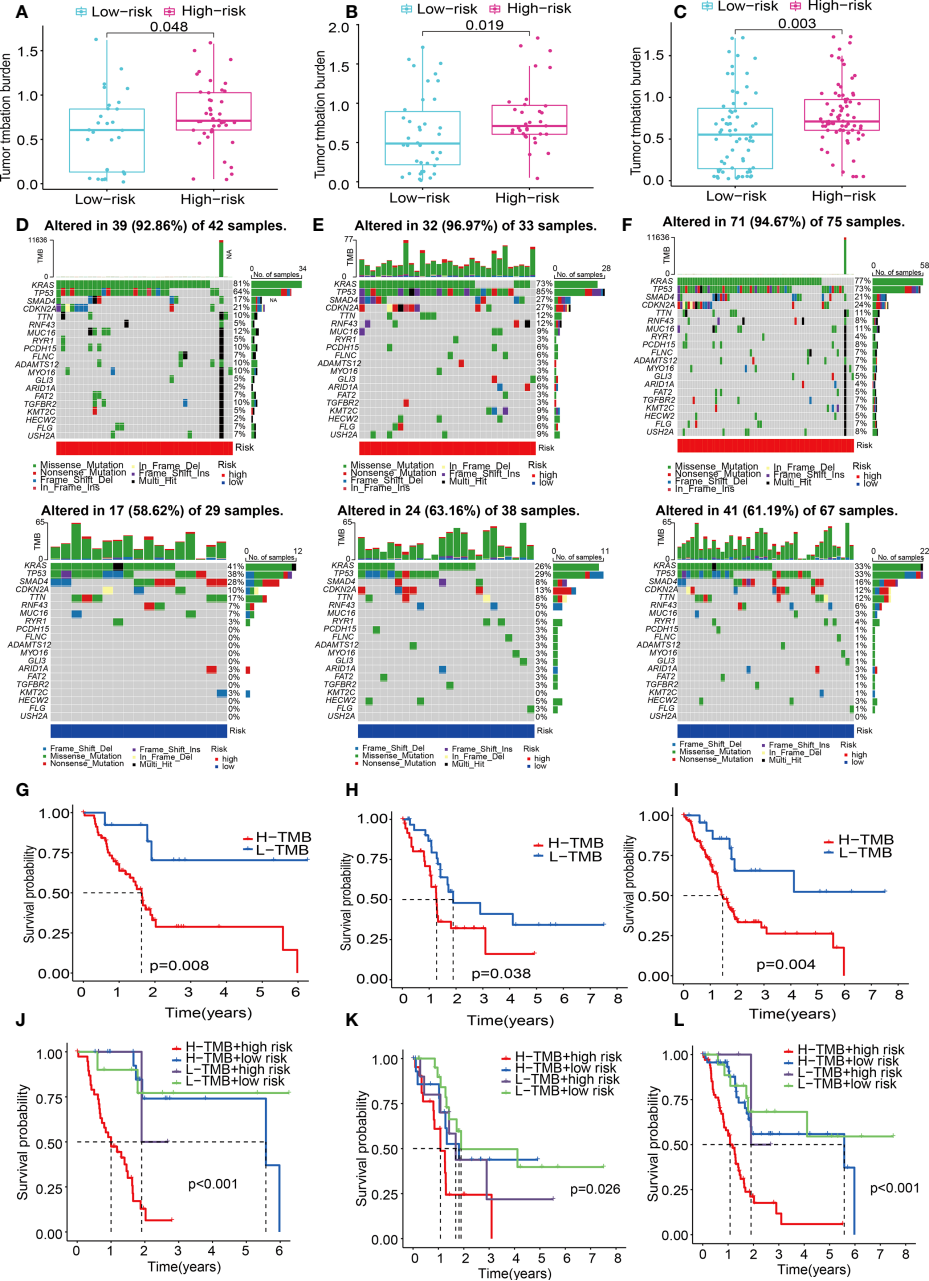
ECM-APP-based analysis of tumor mutation profiles and their clinical correlation. **(A–C)** The discrepancy in the TMB scores between low-risk and high-risk subgroups in the train, test1, and test2 cohorts. **(D–F)** The waterfall plot showed the frequently mutated genes in low-risk and high-risk groupings from the train, test1, and test2 cohorts. High risk is indicated by the red line, while low risk is denoted by the blue line. **(G–I)** Survival analysis of TMB scores in low- and high-risk subgroups from the train, test1, and test2 cohorts. **(J–L)** Survival analysis stratified by the risk scores and TMB scores from the train, test1, and test2 cohorts.

Numerous studies have shown that TMB is linked to elevated CD8+ T cell infiltration levels. These lymphocytes can identify cancer cells and subsequently initiate anticancer responses (28–30). For that, the association between TMB and risk score was explored using the mutation data of PAAD patients in the train, test1, and test2 cohorts. To begin, the TMB levels were discovered in both the low- and the high-risk subgroups. There was a substantial difference in TMB levels between the two subgroups (P < 0.05) in the train, test1, and test2 cohorts (**Figures 12A–C**), and the TMB scores were statistically elevated in the high-risk subgroup. Additionally, we researched and created a visual representation of, the distribution of gene mutations in the low- and high-risk score subgroups. The research evaluated the mutation status and clinical and pathological characteristics of the topmost 20 driver genes that had the most frequent alteration. The mutation counts of genes were visibly elevated in the high-risk subgroup in contrast with those in the low-risk subgroup in the train, test1, and test2 cohorts, principally including KRAS, tumor protein p53 (TP53), and CDKN2A (**Figures 12D–F**).

Patients diagnosed with PAAD who had elevated TMB scores showed a lower OS time as opposed to those who had low TMB values across all three cohorts (**Figures 12G–I**). To gain a deeper comprehension of the effectiveness of the consistent prognostic value of TMB and risk score, we verified the synergistic impact of the two markers in the prognosis prediction of PAAD. Interference of TMB status with the prognosis prediction accuracy of risk scores was observed, as shown by the stratified survival curve. We found patients with high TMB scores and risk scores had a worse survival time than the others in the train, test1, and test2 cohorts (**Figures 12J–L**). In summary, these data revealed that risk score might serve independently as a predictor and could evaluate the clinical outcome of immunological therapy for anti-tumor treatment.

### Characterization of tumor immune microenvironment between low-risk and high-risk populations

**Figure 13:**
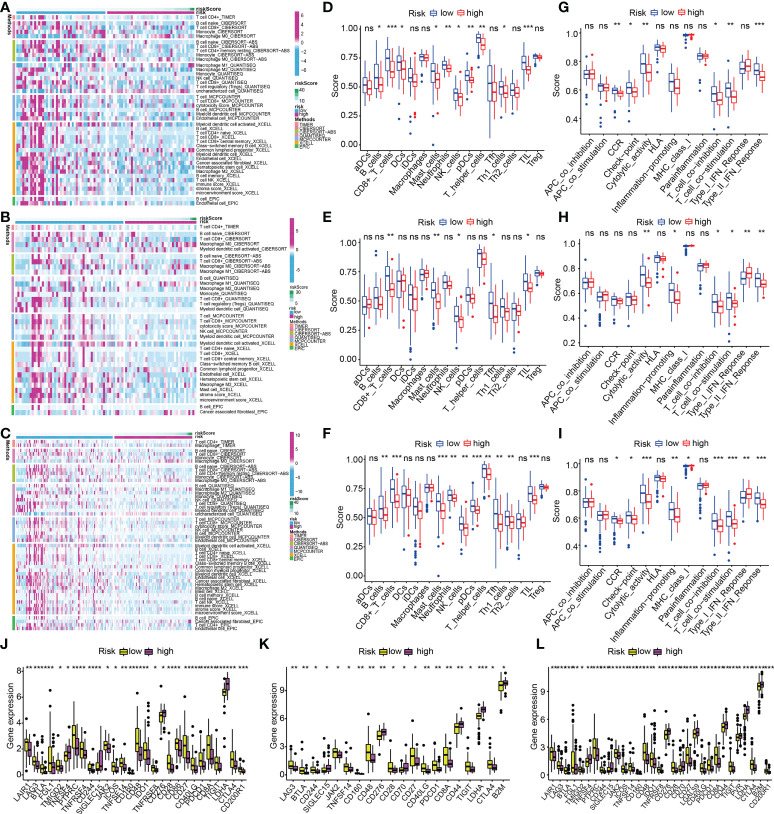
ECM-APP-based analysis of tumor immune microenvironment. **(A–C)** The heat maps showed discrepancies in infiltration levels of immune cells across low- and high-risk subgroups premised on the train, test1, and test2 cohorts. The box plots showed discrepancies in ssGSEA scores between two risk subgroups in the **(D, G)** train cohort, **(E, H)** test1, and **(F, I)** test2cohorts. **(J–L)** The box plots showed discrepancies in immune checkpoint expression in low- and high-risk subgroups premised on the train, test1, and test2 cohorts. (* indicates p <0.05; ** indicates p < 0.01; *** indicates p < 0.001. ns, non-significant).

The variation of immunocyte infiltration between low- and high-risk was calculated by EPIC, XCELL, MCPCOUNTER, QUANTISEQ, CIBERSORT-ABS, CIBERSORT, and TIMER algorithms. The heat map about immune cell infiltration showed most immune cells had fewer infiltrations in the high-risk subgroup in the train cohort (**Figure 13A**), which was validated by the findings of the infiltration status of immune cells in test1 and test2 cohorts (**Figures 13B, C**). To get a deeper comprehension of the connection between the risk score and the immune state, we performed ssGSEA to quantify the enrichment scores of a variety of immune cell subpopulations and the functions that are associated with them. The components that make up the antigen presentation process, notably the score of B cells, T cells, DCs, mast cells, neutrophils, NK cells, pDCs, Type II IFN response, T cell co−stimulation, Th1 cells, T cell co−inhibition, TIL, cytolytic activity, check-point, CCR, T helper cells, were significantly different across the high- and low-risk subgroups in the train cohort (all adjusted P< 0.05, **Figures 13D, G**). Interestingly, we found that the score of all the immune cells, as well as immune-associated functions, were lower in the high-risk subgroup, which could be confirmed in the test1(**Figures 13E, H**) and test2 cohorts(**Figures 13F, I**). Considering the importance of immune checkpoints for immune cells to exert their anti-tumor function, the gene expressions of immune checkpoint-related molecules between high- and low-risk subgroups were depicted in **Figure 13J**. As we could see, all the immune checkpoint-related molecules had significantly statistical differences in the train cohort. The results of immune checkpoints in test1 and test2 cohorts were consistent with this (**Figures 13K, L**). And in the low-risk subgroups, the gene expressions of immune checkpoint-related molecules were higher than in high-risk subgroups.

## Discussion

PAAD is characterized by an extraordinarily dense fibrotic stroma, which hinders both the perfusion of the tumor as well as the delivery of anticancer medications (31). Because the ECM comprises the vast majority of the stroma, it is the primary factor in the stroma’s elevated interstitial tissue pressure as well as its stiff mechanical characteristics (32). Besides its mechanical effect, the ECM also provides critical physical and biochemical signals that stimulate the survival, proliferation, and metastases of cancer cells (33). Furthermore, PAAD cells can endure the nutrient-deficient environment due to the ECM, which acts as a source of nutrients for the cells (33). Even though treatment approaches utilizing stroma-depleting medications have shown unsatisfactory results, a growing body of research suggests that the ECM might provide several viable therapeutic targets (32). Thus, ECM-based risk stratification of PAAD is a promising strategy for prognosis assessment and individual management.

First, a comprehensive investigation of the pan-cancer-related ECMGs was carried out with the assistance of a wide variety of bioinformatics-related technologies. For the first time, the expression traits, prognostic values, methylation levels, mutation profiles, and signaling pathway correlation of ECMGs in pan-cancer have been presented using heatmaps, which opens up a wide variety of possible study avenues for the investigation of ECMGs in the future. Because PAAD was the primary focus of our work, we devoted more time and effort so as we can thoroughly analyze it. We found that the majority of ECMGs were present as risky genes in the patients who had PAAD. This finding was in line with the findings from the earlier studies, which showed that upregulation of ECMGs may contribute to the progression of cancer (4). Of note, our findings also revealed that ITGA7, CD36, SDC3, COL4A6, and TNXB functioned as protective genes in PAAD, which was an inconsistent phenomenon that ITGA7 and CD36 had been reported to serve as risky genes in the onset, progression, and chemoresistance of PAAD (34, 35). It’s conceivable that these contradicting findings are because there are still pathways that haven’t been found, and they might be competing with each other for dominance or indicate heterogeneous tumors.

It has been well-established that genes with both differential expression traits and clinical significance could be served as the hub genes of the occurrence and progression of diseases. Our findings illustrated considerable variations in the expression of COL11A1, ITGA3, ITGB4, ITGB6, LAMA3, LAMB3, and LAMC2 between PAAD and normal pancreatic tissues, which was verified by qRT-PCR and IHC. Furthermore, above 7 ECMGs were also detected to be significantly associated with prognosis and stage of PAAD, indicating their crucial roles in the pathophysiologic mechanisms of PAAD.

Subsequently, we further stratified patients with PAAD into 3 clusters depending on their ECMG expression levels and ECM scores. The OS rates of patients who belonged to the ECM-inactive cluster were reported to be substantially greater in contrast with those of patients who belonged to the ECM-active cluster, demonstrating that genes implicated in ECM were mostly risky.

The predominant therapies for PAAD are those that target the ECM pathway, including poly ADP-ribose polymerase inhibitors, tyrosine kinase inhibitors, and plant anticancer drugs (36, 37). Therefore, in the treatment of PAAD, we investigated the potential involvement of some of the most widely administered medicines that target ECMGs. We discovered that the three patient clusters had distinct responses to the medications that were investigated, which suggests that patients may receive a treatment plan that is better tailored to their specific needs if ECMG expression profiles were used to make the decision. For example, patients whose ECM pathway is inactive may benefit more from treatment with gefitinib and AZD.8055 (mTOR Inhibitor), while patients with highly active ECM pathways may gain more benefit from treatment with AZD.0530 (saracatinib) and docetaxel.

It is now a well-established and consensually-acknowledged fact that the aberrant expression of oncogenes and tumor suppressor genes might result in the onset and progression of PAAD. ECM might disrupt the balance between tumor suppressor genes and oncogenes, thus aggravating the condition and worsening the prognosis of PAAD. Surabhi and his colleagues reported that the collagen milieu promotes resistance to chemotherapy in PAAD via elevated histone H3K9 and H3K27 acetylation levels, as well as elevated expression levels of GCN5, PCAF, and p300 histone acetyltransferases, which indicated that ECM exerts profound epigenetic effects on PAAD cells (38). Similarly, our findings revealed that the expression levels of several classical oncogenes and histone modification-related genes (i.e. sirtuins (SIRTs) and histone deacetylases (HDACs)) across the three ECM-based clusters were significantly different. For instance, the decreased levels of oncogenes expression (MTOR, KRAS, MYC, CCND1, PIK3CA, MUC16, HDAC1, HDAC7, and HDAC9) and increased expression levels of tumor suppressors (SIRT3 and SIRT6) in the C2 populations might account for their poor prognoses.

The infiltrating immune cells are a significant part of human malignancies. It progressively became apparent that most neoplasms were not considered foreign to the host and that specific infiltration of immune cells exhibited the potential to promote tumor progression and metastasis, and the immune cells are intimately linked to the clinical outcomes of neoplasms, which could be considered as the effective targets for anticancer treatment (25, 39, 40). Therefore, we assessed the connection between the factors related to the infiltration levels of immune cells and the ECMGs in our study. We found that ECM performs a fundamental function in immune suppression, and has the potential to induce primary as well as secondary resistance to immune checkpoint inhibitors. C2 subtype (ECM-inactive) was accompanied by a low expression of immune checkpoints, suggesting its enhanced anti-tumor immune response. This illustrates that it has better clinical outcomes. Treg cells and mast cells may aid in the progression of a tumor by inhibiting the host’s antitumor immune responses (41, 42). Additionally, the T helper cell might promote tumor metastasis, leading to a dismal prognosis in pancreatic cancer patients (43). As a result, the findings of the association between the ECM score and the mast cells, Treg cells, and T helper cells were in line with our hypothesis. The abundance of the above-mentioned cancer-promoting immune cells positively correlated to ECM score, which might account for the poor prognosis in the C1 subtype.

After that, we developed a risk panel by employing LASSO-Cox regression analysis to predict the patients’ chances of surviving PAAD. K-M analysis and ROC curves were employed in the train cohorts, the internal validation cohort, and the external validation cohort to confirm the outstanding prognostic ability of our ECM-APP. Our ECM-APP was shown to independently serve as a prognostic marker in PAAD utilizing, as evidenced by univariate and multivariate Cox regression analyses.

TMB is a new biological marker for predicting the clinical outcomes of PAAD patients as well as their responsiveness to immunotherapeutic treatment (44). The waterfall plot revealed that the mutation counts of genes were visibly elevated in the high-risk subgroup in contrast with those in the low-risk subgroup, especially for KRAS and TP53. Following that, a stratified survival curve was generated, which revealed that the risk score had prognostic predictive potential, which was independent of TMB, indicating that risk score and TMB characterize diverse aspects of immunology. In addition, the risk score in conjunction with the mutation data showed a substantial variation in the frequency of gene variation between the high- and the low-risk subgroups when viewed from the perspective of the transcriptome.

Because immune infiltration served as a significant driving element in PAAD, we continued our investigation into the fundamental roles played by the tumor immune microenvironment in our ECM-APP. Our data showed that low-risk populations were accompanied by a multitude of anti-tumor immune cell infiltration (e.g. CD4+ T cells, B cells, CD8+ T cells, and NK cells), whereas high-risk populations were accompanied by a multitude of M0 macrophage infiltration. These findings suggested that the high-risk subgroup was an immunosuppressive phenotype, whereas the low-risk subgroup was characterized by an exuberant anti-tumor immune response. Additionally, the expression of immune checkpoints showed a significant difference between low-risk and low-risk populations, which was also one of the possible causes for different clinical outcomes.

## Conclusion

Through integrating a series of bioinformatics methods, seven hub ECMGs (i.e. LAMB3, LAMA3, ITGB6, ITGB4, ITGA2, LAMC2, and COL11A1) that has the potential to act as a new indicator of both the advancement and prognosis of PAAD were identified *via* our research. The pathogenesis of PAAD was associated with an abnormally activated ECM-receptor interaction. Patients with PAAD could be classified into three clusters (i.e. ECM-inactive, ECM-normal, and ECM-active) with different prognosis, immune characteristics, and drug sensitivity based on ECM scores. A novel ECM-APP with excellent prediction capacity was created and verified, which may offer an attractive strategy to predict PAAD patients’ prognoses.

## Data availability statement

The original contributions presented in the study are included in the article/**Supplementary Material**. Further inquiries can be directed to the corresponding authors.

## Ethics statement

The studies involving human participants were reviewed and approved by Ethics Committee of the First Affiliated Hospital of Dalian Medical University (ID: PJ-KS-KY-2022-60). The patients/participants provided their written informed consent to participate in this study.

## Author contributions

XC, QY, and JL are the co-first authors. XC, QY, and JL were responsible for the design of the research methods, the execution of the experiments, the analysis of the data, as well as the drafting and revision of the manuscript. DS, SL, and ZW conceived the research methodologies, and revised the manuscript. SX, XS, and YS assisted with the gathering of data and conducting the tests. XC also collected the clinical samples. All authors contributed to the article and approved the submitted version.

## Funding

The research was conducted with support from the Leading Talent Team of Support Program for High Level Talent’s Innovation of Dalian in 2019 (2019RD11), the Key Projects of Overseas Training Foundation of the Higher Education Institutions of Liaoning Province, China (2020GJWZD004), and the National Natural Science Foundation of China (No. 82000075).

## Acknowledgments

We thank Bullet Edits Limited for the linguistic editing of the manuscript.

## Conflict of interest

The authors declare that the research was conducted in the absence of any commercial or financial relationships that could be construed as a potential conflict of interest.

## Publisher’s note

All claims expressed in this article are solely those of the authors and do not necessarily represent those of their affiliated organizations, or those of the publisher, the editors and the reviewers. Any product that may be evaluated in this article, or claim that may be made by its manufacturer, is not guaranteed or endorsed by the publisher.

## Supplementary material

Supplementary Figure 1 Analysis of the single-cell types atlas of seven hub ECMGs **(A)**: COL11A1; **(B)**: LAMC2; **(C)**: LAMA3; **(D)**: LAMB3; **(E)**: ITGA2; **(F)**: ITGB4; **(G)**: ITGB6) related to the prognosis and stage of PAAD by HPA database.

Supplementary Figure 2 Determination of differentially expressed ECM-associated mRNAs and lncRNAs with prognostic significance. **(A)** Identification of 278 ECM-related lncRNAs. The correlation analysis between 83 ECM-related mRNAs and 278 lncRNAs was performed by Pearson correlation analysis (|R| >0.4 and P<0.01). **(B)** Univariate Cox regression analysis showed the prognostic values of 149 differentially expressed ECM-related lncRNAs and mRNAs in PAAD. **(C)** The LASSO coefficient profiles of ECM-related lncRNAs and mRNAs in PAAD. **(D)** The cross-validation results of model construction. A total of nine genes were filtered by LASSO Cox regression analysis. **(E)** Multivariate Cox regression analysis of nine ECM-related mRNAs and lncRNAs.
