# Supplementary material for: Comprehensive characterization of extracellular matrix-related genes in PAAD identified a novel prognostic panel related to clinical outcomes and immune microenvironment: A silico analysis with in vivo and vitro validation
Source: Front Immunol. 2022 Oct 13;13:985911. doi: 10.3389/fimmu.2022.985911 (PMC9606578; doi:10.3389/fimmu.2022.985911)
Supplement: Supplementary file 3 [file Table_3.docx]

**Abbreviations**

ECM: Extracellular matrix; ECMGs: ECM-related genes; PAAD: pancreatic adenocarcinoma; ECM-APP: ECM-associated mRNA-lncRNA-based prognostic panel; TME: Tumor microenvironment; OS: Overall survival; ssGSEA: Single sample gene set enrichment analysis; GSEA: Gene set enrichment analysis; GEO: Gene Expression Omnibus; GTEx: Genotype-Tissue Expression; ICGC: International Cancer Genome Consortium; TCGA: The Cancer Genome Atlas; GEPIA: Gene Expression Profiling Interactive Analysis; IHC: immunohistochemistry; qRT-PCR: Quantitative real-time PCR; (ATCC, Manassas, VA, USA): The American Type Culture Collection; DMEM: Dulbecco's modified Eagle's medium; FBS: Fetal bovine serum; IMDM: Iscove's Modified Dulbecco medium; HPA: Human Protein Atlas; GDSC: Genomics of Drug Sensitivity in Cancer; SIRTs: Sirtuins; HDACs: Histone deacetylases; ICGs: immune checkpoints genes; Treg: Regulatory T cells; LncRNAs: Long non-coding RNAs; Gtf: Gene transfer format; LASSO: Least absolute shrinkage and selection operator; AUC: Area under the curve; ROC: Receiver-operating characteristic; TMB: Tumor mutation burden; CNV: Copy number variations; SNV: Single-nucleotide variant; UCEC: Uterine corpus endometrial carcinoma; SKCM: Skin cutaneous melanoma; STAD: Stomach adenocarcinoma; COAD: Colon adenocarcinoma; LUSC: Lung squamous cell carcinoma; LUAD: Lung adenocarcinoma; UCS: Uterine carcinosarcoma; OV: Ovarian serous cystadenocarcinoma; SARC: Sarcoma; KIRC: Kidney renal clear cell carcinoma; TGCT: Testicular germ cell tumors; PRAD: Prostate adenocarcinoma; ESCA: Esophageal carcinoma; PAAD: Pancreatic adenocarcinoma; BLCA: Bladder urothelial carcinoma; LGG: Brain lower-grade glioma; CESC: Cervical squamous cell carcinoma and endocervical adenocarcinoma; LIHC: Liver hepatocellular carcinoma; DLBC: Lymphoid neoplasm diffuse large B-cell lymphoma; MESO: Mesothelioma; KIRP: Kidney renal papillary cell carcinoma; THYM: Thymoma; HNSC: Head and neck squamous cell carcinoma; GBM: Glioblastoma multiforme; LAML: Acute myeloid leukemia; UVM: Uveal melanoma; READ: Rectum adenocarcinoma; PCPG: Pheochromocytoma and paraganglioma; ACC: Adrenocortical carcinoma; CHOL: Cholangiocarcinoma; THCA: Thyroid carcinoma; KICH: Kidney chromophobe; BRCA: Breast invasive carcinoma; mTOR: Mammalian target of rapamycin; IC50: Half maximal inhibitory concentration; HR: Hazard ratio; DCs: Dendritic cells; NK cells: Natural killer cells; pDCs: Plasmacytoid dendritic cells; TIL: Tumor infiltrating lymphocytes; CCR: Cytokine cytokine receptors; K-M: Kaplan-Meier;
