# Supplementary material for: Comprehensive characterization of extracellular matrix-related genes in PAAD identified a novel prognostic panel related to clinical outcomes and immune microenvironment: A silico analysis with in vivo and vitro validation
Source: Front Immunol. 2022 Oct 13;13:985911. doi: 10.3389/fimmu.2022.985911 (PMC9606578; doi:10.3389/fimmu.2022.985911)
Supplement: Supplementary file 4 [file Table_4.docx]

The size of gene expression profiles is too large, so we have uploaded it into the public website. Of note, the expression matrix was changed into log transformation and corrected by the “sva” package. All the scholars can use our uploaded data to re-visualize all the results of this research. The downloaded link is as follows:

https://www.jianguoyun.com/p/DbrdNmAQtf_aChjVltoEIAA
