# Supplementary figures and images for: Comprehensive characterization of extracellular matrix-related genes in PAAD identified a novel prognostic panel related to clinical outcomes and immune microenvironment: A silico analysis with in vivo and vitro validation
Source: Front Immunol. 2022 Oct 13;13:985911. doi: 10.3389/fimmu.2022.985911 (PMC9606578; doi:10.3389/fimmu.2022.985911)

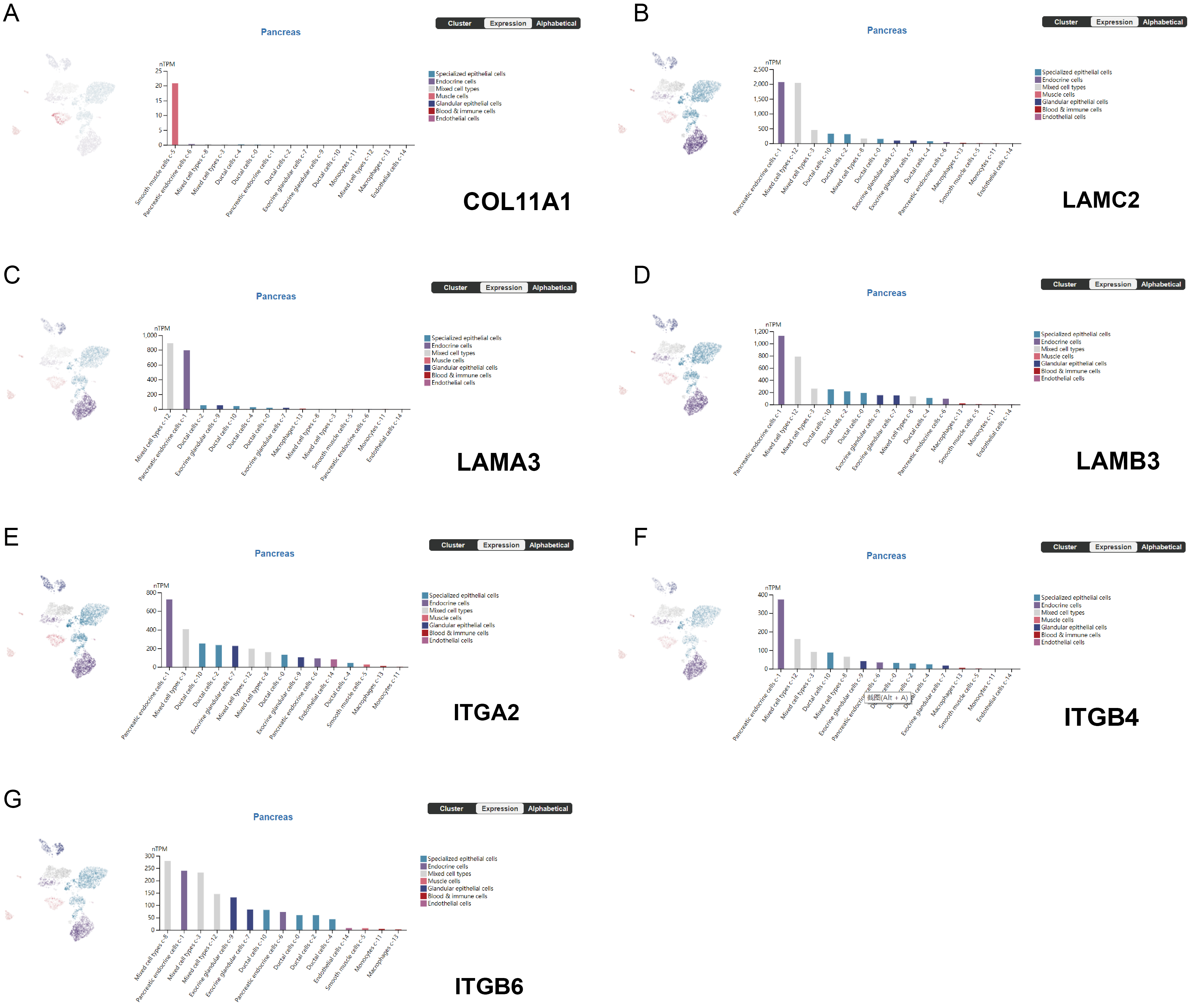

Supplement: Supplementary Figure 1 — Analysis of the single-cell types atlas of seven hub ECMGs (A): COL11A1; (B): LAMC2; (C): LAMA3; (D): LAMB3; (E): ITGA2; (F): ITGB4; (G): ITGB6) related to the prognosis and stage of PAAD by HPA database. [file Image_1.jpeg]

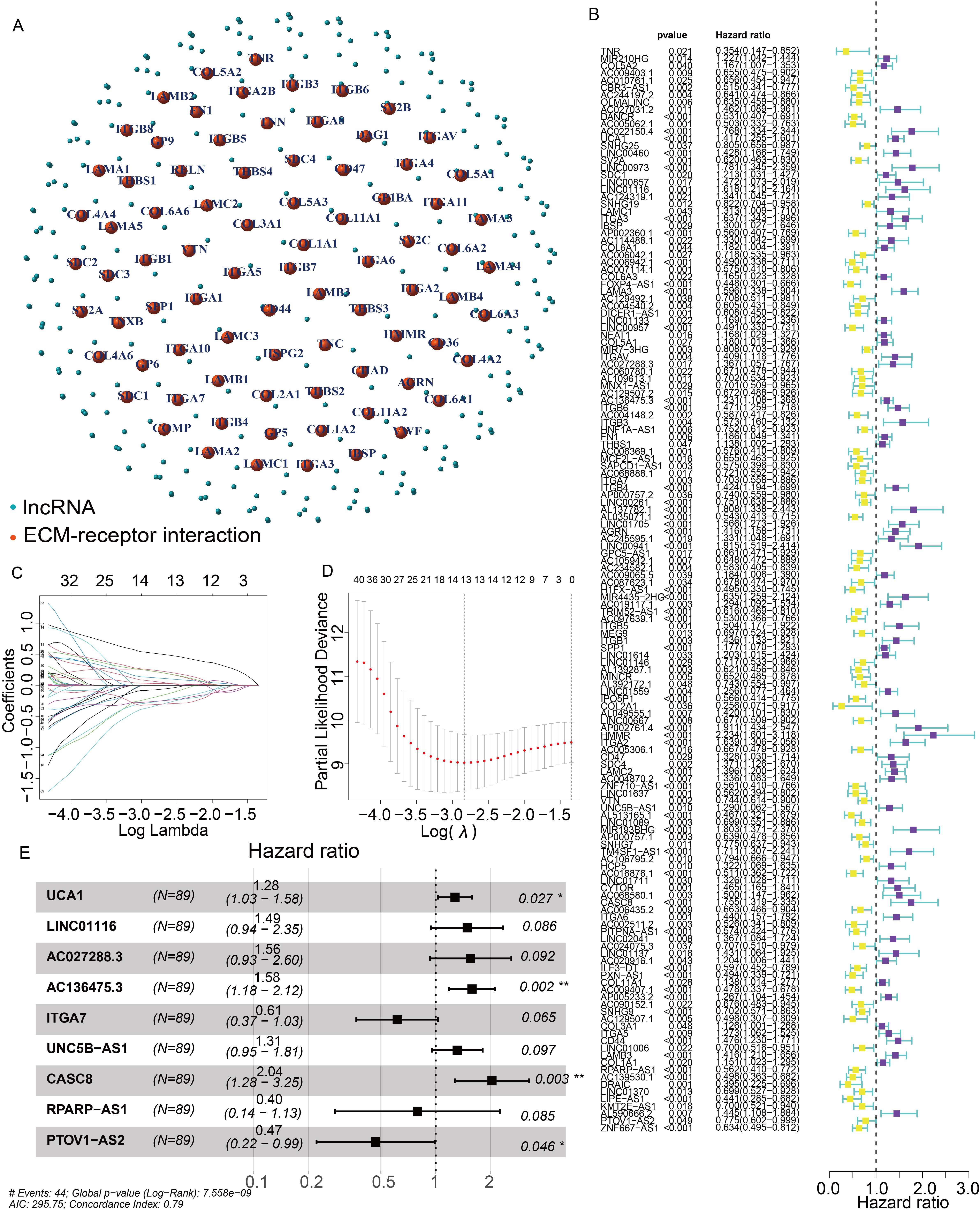

Supplement: Supplementary Figure 2 — Determination of differentially expressed ECM-associated mRNAs and lncRNAs with prognostic significance. (A) Identification of 278 ECM-related lncRNAs. The correlation analysis between 83 ECM-related mRNAs and 278 lncRNAs was performed by Pearson correlation analysis (|R| >0.4 and P<0.01). (B) Univariate Cox regression analysis showed the prognostic values of 149 differentially expressed ECM-related lncRNAs and mRNAs in PAAD. (C) The LASSO coefficient profiles of ECM-related lncRNAs and mRNAs in PAAD. (D) The cross-validation results of model construction. A total of nine genes were filtered by LASSO Cox regression analysis. (E) Multivariate Cox regression analysis of nine ECM-related mRNAs and lncRNAs. [file Image_2.jpeg]
